# Optimal Management of the Unilateral Recurrent Laryngeal Nerve Involvement in Patients with Thyroid Cancer

**DOI:** 10.3390/cancers13092129

**Published:** 2021-04-28

**Authors:** Satoru Miyamaru, Daizo Murakami, Kohei Nishimoto, Narihiro Kodama, Joji Tashiro, Yusuke Miyamoto, Haruki Saito, Hiroki Takeda, Momoko Ise, Yorihisa Orita

**Affiliations:** 1 Department of Otolaryngology-Head and Neck Surgery, Graduate School of Medicine, Kumamoto University, Kumamoto 860-8555, Japan; daizo-m@kuh.kumamoto-u.ac.jp (D.M.); koheihei@kuh.kumamoto-u.ac.jp (K.N.); tashiro.joji@kuh.kumamoto-u.ac.jp (J.T.); mymtyusk@kuh.kumamoto-u.ac.jp (Y.M.); sh91385@kuh.kumamoto-u.ac.jp (H.S.); takeda.hiroki@kuh.kumamoto-u.ac.jp (H.T.); ise-m@kuh.kumamoto-u.ac.jp (M.I.); yoriorita@kumamoto-u.ac.jp (Y.O.); 2 Department of Rehabilitation, Kumamoto Health Science University, Kumamoto 860-8556, Japan; kodama@kumamoto-hsu.ac.jp

**Keywords:** thyroid cancer, recurrent laryngeal nerve, vocal fold, vocal function, recurrent laryngeal nerve reconstruction

## Abstract

**Simple Summary:**

Recurrent laryngeal nerve (RLN) is the second most common structure invaded by primary or metastatic thyroid cancer. However, little is known about the optimal procedure for maintaining vocal function in patients with unilateral RLN involvement in thyroid cancer. This study aimed to evaluate various parameters of vocal function to establish the optimal management of thyroid cancer patients with unilateral RLN involvement. Based on our findings, we propose that for optimal management of unilateral RLN involvement in thyroid cancer, first, sharp dissection should be performed, and if this is impossible, a simultaneous RLN reconstruction procedure should be adopted whenever possible. These findings may help improve management of RLN involvement in patients with thyroid cancer and ensure vocal function preservation.

**Abstract:**

We aimed to determine the optimal management of recurrent laryngeal nerve (RLN) involvement in thyroid cancer. We enrolled 80 patients with unilateral RLN involvement in thyroid cancer between 2000 and 2016. Eleven patients with preoperatively functional vocal folds (VFs) underwent sharp tumor resection to preserve the RLN (shaving group). Thirty-three patients underwent RLN reconstruction with RLN resection (reconstruction group). We divided the reconstruction group into two subgroups based on preoperative VF mobility (normal-reconstruction and paralyzed-reconstruction subgroups). In the cases where RLN reconstruction was difficult, phonosurgeries including arytenoid adduction (AA), with or without thyroplasty type I, or nerve muscle pedicle implantation with AA were performed later (phonosurgery group). We evaluated and compared vocal function among the evaluated periods and different groups. Postoperative vocal function in the shaving and normal-reconstruction subgroups was favorable. There were no significant differences between the two groups. In the paralyzed-reconstruction and phonosurgery groups, postoperative vocal function was significantly improved, and vocal function in the paralyzed-reconstruction subgroup was significantly better than that in the phonosurgery group. For optimal management of unilateral RLN involvement in thyroid cancer, first, sharp dissection should be performed, and if this is impossible, a simultaneous RLN reconstruction procedure should be adopted whenever possible.

## 1. Introduction

After the strap muscles, the recurrent laryngeal nerve (RLN) is the second most common structure invaded by primary or metastatic thyroid cancer [1]. RLN paralysis (RLNP) can cause persistent breathy hoarseness, shortening of phonation, and aspiration, which adversely affects patient quality of life [2,3]. Therefore, in thyroid cancer patients with preoperatively functional vocal folds (VF), first, sharp tumor resection from the RLN should be considered to maintain good voice and VF mobility with oncological safety [4,5,6,7,8]. When it is difficult to preserve the RLN during complete removal of the thyroid cancer, it is desirable to adopt the RLN reconstruction procedure simultaneously during thyroid surgeries [9,10,11,12,13]. Restoration of the muscle bulk and tension due to nerve reinnervation leads to excellent vocal outcomes. Some methods for RLN reconstruction have been reported, including direct RLN anastomosis [14,15], graft interposition between the dissected edges of the RLN [16], and ansa cervicalis nerve (ACN) to RLN anastomosis [17,18]. When RLN reconstruction is difficult, phonosurgeries, such as thyroplasty type I [19,20], arytenoid adduction (AA) [21], VF injection augmentation [22], or nerve muscle pedicle (NMP) transplantation [23,24,25,26,27,28], should be considered. NMP transplantation is a method to induce reinnervation of the laryngeal muscle with implantation of other muscle nerve branches to the laryngeal muscle directly.

However, there are few reports describing the optimal procedure for maintaining vocal function in patients with unilateral RLN involvement in thyroid cancer. In the present study, various parameters of vocal function were analyzed to establish the optimal management of thyroid cancer patients with unilateral RLN involvement.

## 2. Materials and Methods

During a 17-year period from 2000 through 2016, we managed 80 patients with unilateral RLN involvement by thyroid cancer at Kumamoto University Hospital, Kumamoto, Japan, which is the tertiary oncology referral center in the area. Written informed consent was obtained from all patients. This research was approved by the ethical committee for medical and health research involving human subjects at Kumamoto University Hospital (No. 2338). Of the 80 patients, 15 were men (19%) and 65 women (81%), and they were aged 35–79 years (median age, 58 years). Of the 80 patients, 11 with preoperatively functional VF underwent preservation of the RLN involved in thyroid cancer using sharp dissection (shaving group). Thirty-three patients underwent immediate RLN reconstruction after resection of the RLN during thyroid cancer surgery (reconstruction group). The reconstruction group was further divided into two subgroups based on their preoperative VF mobility. Patients with preoperatively functional VF who needed RLN resection and reconstruction were named the normal-reconstruction subgroup (*n* = 12), and those with preoperatively paralyzed VF who underwent RLN resection and reconstruction were referred to as the paralyzed-reconstruction subgroup (*n* = 21). For the RLN reconstruction procedure, we selected direct RLN anastomosis (direct anastomosis, *n* = 4), graft interposition between the dissected edges of the RLN (nerve interposition, *n* = 12), and ACN to RLN anastomosis (nerve transfer, *n* = 17). The remaining 36 patients underwent phonosurgeries to persistent unilateral RLNP for some time after thyroid surgery (phonosurgery group). This group included patients who underwent thyroid surgery at other hospitals. The procedures adopted for the 36 patients included NMP implantation with AA (*n* = 20), and AA with or without thyroplasty type I (*n* = 16). Table 1 summarizes the characteristics of all patients in this study.

**Figure 1:**
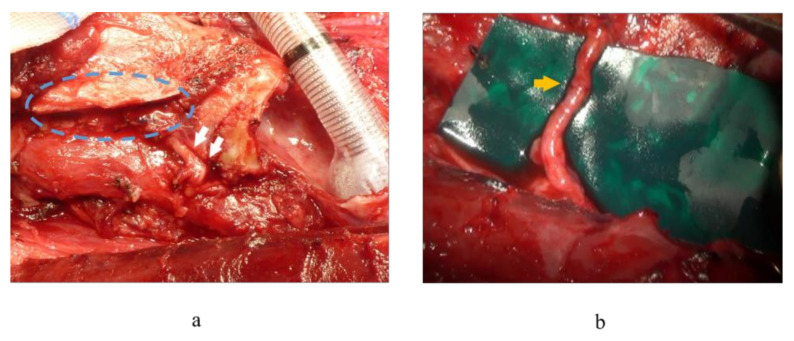
(**a**) Intraoperative findings after resection of the right recurrent laryngeal nerve (RLN), thyroid cartilage (*broken circle*), and inferior pharyngeal constrictor muscle with thyroid cancer. The distal edge of the resected RLN (*arrow*) was secured for the RLN reconstruction procedure. Head to the left. (**b**) Intraoperative finding of nerve transfer between the right RLN and the ansa cervicalis nerve (*arrow*). Head to the left.

**Figure 2:**
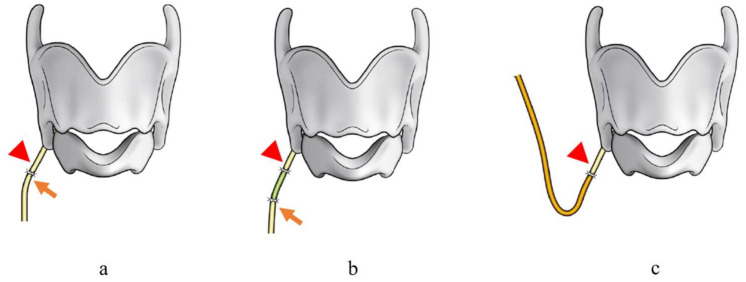
Recurrent laryngeal nerve (RLN) reconstruction procedures. Arrows show proximal edges of right RLN. Arrow heads show distal edges of right RLN: (**a**) direct anastomosis, (**b**) nerve interposition, (**c**) nerve transfer (Citation: Ref. [30]).

**Figure 3:**
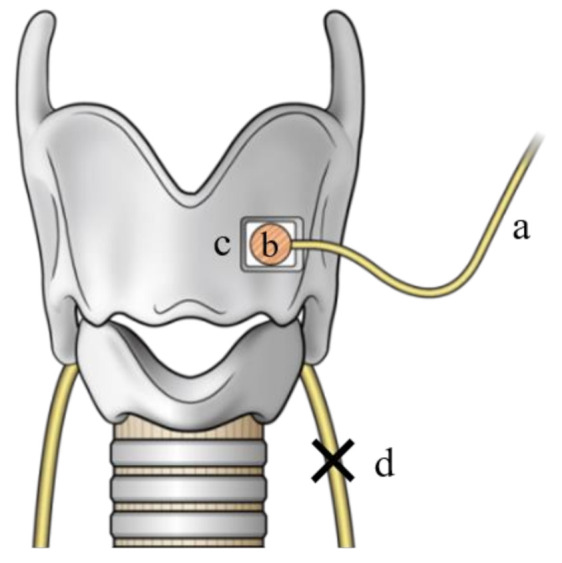
Nerve muscle pedicle (NMP) transplantation method. An NMP flap was made using an ansa cervicalis nerve branch (**a**) and a piece of the sternohyoid muscle (**b**); a window was opened in the thyroid cartilage (**c**) to expose the laryngeal muscle bundle; then, the NMP flap was securely implanted onto the laryngeal muscle through the window under microscopic guidance; (**d**) resected left recurrent laryngeal nerve (Citation: Ref. [30]).

**Figure 4:**
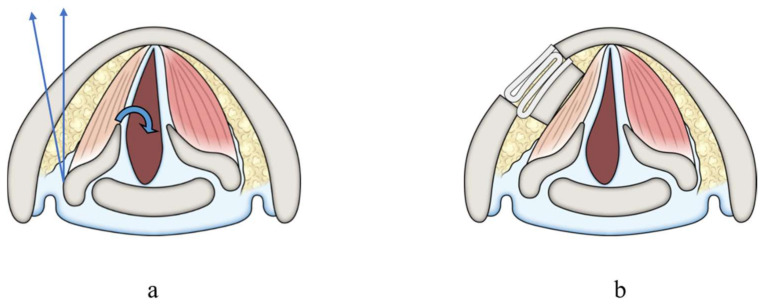
Laryngeal framework surgeries. Laryngeal images of axial plane. (**a**) Arytenoid adduction. We pulled arytenoid cartilage in the direction of the linear arrows, then the vocal fold was rotated at the same median position as the curved arrow. (**b**) Thyroplasty type I. We used a Gore-Tex sheet to push the vocal fold medially through a window opened in the thyroid cartilage. (Citation: Ref. [30]).

Our treatment strategies were as follows. When preoperative VF mobility was normal, we first tried to preserve the RLN involved in thyroid cancer with sharp dissection, which we called the ‘shaving technique’, according to Japanese guidelines for the treatment of thyroid tumor. The tumor was carefully shaved from the RLN using a scalpel under magnification. In cases with signs of invasion into the nerve fiber, such as color alteration, or when the entire circumference of the nerve was surrounded by the tumor, the RLN was extirpated together with the tumor, and RLN reconstruction procedures were performed immediately if it was possible to use the distal stump of the resected RLN, as suggested by Japanese guidelines. If the tumor invaded the RLN at the peripheral portion, and it was difficult to secure adequate nerve length for the reconstruction procedure, we divided the inferior pharyngeal constrictor muscle along the lateral edge of the thyroid cartilage before resecting the nerve to identify it under the muscle [29]. In cases where the tumor invaded more of the peripheral portion of the RLN, we dissected the inferior horn or lateral plane of the thyroid cartilage to identify the nerve located by the side of the thyroid cartilage (Figure 1a,b). With regard to the RLN reconstruction procedure, we first attempted direct anastomosis if this was possible (direct anastomosis, Figure 2a). When the length of the resected RLN was too long to perform direct anastomosis, free nerve graft implantation between the dissected edges (nerve interposition: Figure 2b) or ACN to RLN anastomosis (nerve transfer: Figure 2c) were performed. The nerve graft for interposition was usually acquired from the great auricular nerve or the ACN. When nerve transfer was performed, the ACN was identified at the surface of the internal jugular vein, and its branches to the sternohyoid (SH) muscles were dissected. The major branch, or usually the common branch to these branches, was transected, and the proximal edge was anastomosed to the distal stump of the RLN. In cases where the simultaneous reconstruction of the RLN was difficult because the proximal stumps of the RLN could not be utilized for nerve reconstruction due to extensive tumor infiltration, we considered phonosurgery to persistent unilateral RLNP later, while holding discussions with the patients. Similarly, in cases where excision of the RLN had been performed at other hospitals, we generally chose phonosurgery rather than nerve reconstruction procedures, because it may have been difficult to detect the resected peripheral edge of the RLN, and preservation of CAN was uncertain. For phonosurgery, we first performed NMP with AA under general anesthesia, as previously reported [24] (Figure 3). If it was difficult to use ACN branches for the NMP procedure or if the patients could not undergo general anesthesia for a general condition, we applied AA with or without thyroplasty type I (Figure 4a,b). We performed the operation under local anesthesia to finely tune the procedures by hearing the patient’s voice. During these surgical procedures, we use a neuromonitoring system to identify nerves and confirm the function.

In this study, we assessed vocal function using aerodynamic, acoustic, and perceptual analyses. The patients were asked to undergo vocal function tests preoperatively and at three different postoperative periods: 1, 6, and 12 months after surgery.

### 2.1. Aerodynamic Analysis

For aerodynamic analyses, patients were instructed to produce sustained phonation of the vowel /a/ for as long as possible at a comfortable pitch and loudness. The maximum phonation time (MPT) was measured twice for each patient using a stopwatch, and the greater value was recorded. The mean airflow rate (MFR) was measured using a phonatory function analyzer (PS-77E; Nagashima, Tokyo, Japan). Patients were instructed to produce the vowel /a/ at a comfortable pitch and loudness while keeping a mouthpiece around the lips and wearing a nose clip.

### 2.2. Acoustic Analysis

Acoustic analysis was conducted using the Multi-Dimensional Voice Program Model 5105 (version 3.1.7; Kay Elemetrics, Lincoln Park, NJ, USA). The vowel segment was cut from the complete voice sample, and 0.5–1 s from a stable portion of the vowel was trimmed and analyzed. The acoustic parameters included jitter (normal, <1.04%), shimmer (normal, <3.81%), and noise-to-harmonics ratio (NHR; normal, >7.2 dB). The NHR was converted into harmonics-to-noise (HNR) using the equation HNR = 10 × log_10_ (1/NHR) [31]. 

### 2.3. Perceptual Analysis

For perceptual analysis, patient voices were recorded in a sound-treated room using a digital recorder (Model PMD 670; Marantz, Sagamihara, Japan) connected to a microphone (Model WM-421; Panasonic, Yokohama, Japan). The microphone was held 20 cm away from the mouth during the recordings. Recording samples included name, date, and standard text in Japanese, and sustained phonation of the vowel /a/ at a comfortable pitch and loudness. The voice was digitized at 45 kHz through an antialiasing filter and stored in a pulse modulation format. The speech segments of the recordings were used for auditory perceptual analysis. All speech samples were anonymized before assessment according to the Grade overall-Roughness-Breathiness-Asthenia-Strain voice scale (GRBAS) [32] to prevent any possible bias. Three different listeners assessed the randomly arranged recordings (one laryngologist and two speech pathologists). The mean values of grade (G) and breathiness (B) scores assigned by the three judges using a four-point scale (0 = normal, 1 = slight disturbance, 2 = moderate disturbance, 3 = severe disturbance) were calculated, because unilateral RLNP causes breathy hoarseness.

### 2.4. Statistical Analysis

In this study, we compared vocal outcomes between the evaluation periods in the same group and among different groups. First, the time-dependent vocal function was analyzed in each group. Among the reconstruction group, the difference in vocal function between the normal-reconstruction subgroup (*n* = 12) and the paralyzed-reconstruction subgroup (*n* = 21) was evaluated. Additionally, in the paralyzed reconstruction subgroup, we compared vocal function between the different reconstruction methods (nerve transfer (*n* = 13) vs. nerve interposition (*n* = 8)) and the patient age (under 65 years old (*n* = 11) vs. over 65 years (*n* = 10)). Patients who underwent direct anastomosis of the resected RLN were excluded from the analysis due to preoperative normal VF mobility. The vocal function in the normal-reconstruction subgroup (*n* = 12) was compared with that in the shaving group (*n* = 11), and the paralyzed-reconstruction group (*n* = 21) was compared with that of the phonosurgery group (*n* = 36). All measurements are shown as the mean ± standard deviation. The Mann–Whitney U-test was used for statistical analysis (StatView 5.0 for, SAS Institute, Cary, NC, USA). For all parameters, statistical significance was set at *p* < 0.05.

## 3. Results

### 3.1. Time-Dependent Vocal Function in Each Method of Handling the RLN Involved in Thyroid Cancer

#### 3.1.1. Shaving Group (*n* = 11)

Because complete preoperative and postoperative data did not exist in the shaving group, we evaluated the vocal outcomes 6 months after surgery. The average values of MPT and MFR were 19.5 ± 10.2 s and 135 ± 38 mL/s, respectively. Both values were within the normal ranges. The average jitter, shimmer, and HNR values were 1.523 ± 1.65%, 5.865 ± 3.37%, and 7.433 ± 1.65 dB, respectively. The mean G and B scores at the GRBAS scale were 0.50 ± 0.53 and 0.25 ± 0.46, respectively. The acoustic and perceptual parameters reached near-normal levels.

Regarding VF mobility, three patients had normal VF mobility immediately after the operation. The postoperative paralyzed VF in the remaining seven patients recovered several months after the operation. In one patient, VF mobility was not restored. 

#### 3.1.2. Reconstruction Group (*n* = 33)

The values at 6 and 12 months after surgery for most parameters were significantly better than those before surgery and at 1 month after surgery in the paralyzed reconstruction subgroup (Table 2).

Regarding the difference in the reconstruction method, the values of jitter, shimmer, and HNR preoperatively and 1 month after surgery in the nerve interposition group were significantly better than those in the nerve transfer group. However, the significance disappeared at 6 and 12 months postoperatively (Table 3). Concerning the patients’ age and VF mobility, there were no significant differences in all parameters in every postoperative period between the different reconstruction methods (data not shown).

#### 3.1.3. Phonosurgery Group (*n* = 36)

For all parameters, the values at every postoperative period were significantly better than those before surgery (Table 4).

### 3.2. Comparative Study of Each Method for Managing the RLN Involved by the Thyroid Cancer

#### 3.2.1. Shaving vs. Normal-Reconstruction

We compared the values in the shaving and normal-reconstruction subgroups at 12 months postoperatively. There were no significant differences in any of the parameters (Table 5).

#### 3.2.2. Paralyzed-Reconstruction vs. Phonosurgery

All preoperative parameters of the paralyzed reconstruction subgroup were better than those of the phonosurgery group. At 1 month after surgery, the MPT, MFR, G score, and B score were significantly better in the phonosurgery group than in the paralyzed-reconstruction subgroup. However, at 12 months postoperative for jitter, at 6 and 12 months for shimmer, and at 6 months for HNR, the values in the paralyzed-reconstruction subgroup were significantly better than those in the NMP group (see Table 6, which compares the values appearing in Table 2 and Table 4).

## 4. Discussion

During the management of RLN involvement in thyroid cancer, proper methods must be selected depending on the situation. The vocal outcomes after the shaving technique in this study showed excellent results, that is, aerodynamic parameters were in the normal range and acoustic and perceptual parameters reached near-normal levels. The advantages of the shaving technique include not only good vocal function, but also a high frequency of preservation of VF mobility with excellent oncological safety [4,5,6,7,8]. The restoration of VF mobility after the shaving technique has been reported to range between 60–92% [4,6,7,8]. Similarly, in our study, VF mobility was preserved in 10 (91%) of 11 patients. However, once the RLN had been resected, even after the administration of the immediate RLN reconstruction method, VF mobility was never recovered. This is because a certain degree of misalignment between the adductor and abductor nerve fibers may have occurred.

When the shaving technique is difficult due to severe tumor invasion to the RLN, an immediate RLN reconstruction procedure should be performed simultaneously. In this study, compared with the shaving group, there were no significant differences in any of the parameters in the reconstruction group. RLN reconstruction surgery induces nerve reinnervation of the laryngeal muscles, resulting in the prevention of progressive loss of muscle tone and bulk. Additionally, the position of the paralyzed VF was improved due to laryngeal muscle reinnervation. Iwaki et al. [33] evaluated pre-and postoperative VF positions in patients who underwent immediate RLN reconstruction surgery for unilateral RLNP caused by thyroid cancer. The paralyzed VF was fixed in the paramedian position preoperatively. On the other hand, in many patients who had their RLN previously resected, the VF was fixed in the abducted position. The RLN damaged by thyroid cancer may not be completely denervated and can still stimulate the laryngeal muscles. Because the ratio of adductor to abductor motor axons of the RLN is 4:1 [34], a partially paralyzed VF may be adducted and fixed in the paramedian position. After laryngeal reinnervation procedures, because reinnervation of the laryngeal muscle is achieved, the position of the VFs shift to the median or paramedian position even after RLN resection [35,36]. In this study, most parameters were improved significantly at 6 months postoperatively compared with the preoperative data. This means that reinnervation of the laryngeal muscle will be achieved 6 months after the operation.

Concerning the difference in RLN reconstruction methods, a previous animal study indicated that direct RLN anastomosis showed the best performance both histologically and physiologically [37]. In a clinical study, Miyauchi et al. [38] and Yoshioka et al. [39] reported that the vocal outcome of immediate RLN reconstruction after thyroid cancer surgery was not reflected by surgical methods. They compared vocal outcomes between direct anastomosis, nerve interposition, nerve transfer, and vagus to RLN anastomosis using MPT or phonation efficiency index (MPT/vital capacity). In this study, there were significant differences in some parameters at 1 month after surgery between nerve interposition and nerve transfer. However, significant differences in these parameters also existed preoperatively and they disappeared at 6 months or later. It is too early to induce nerve reinnervation of the laryngeal muscles at 1 month after surgery with the RLN reconstruction procedure; the differences would be influenced by the patient backgrounds between the two groups. Additionally, the vocal outcomes after the immediate RLN reconstruction procedure were not affected by patient age or preoperative RLNP in this study. These results are similar to those shown in previous reports [38,39]. Therefore, surgeons should select the easiest and most appropriate methods for RLN reconstruction.

If it is impossible to carry out the RLN reconstruction method simultaneously during thyroid cancer surgery, we have to perform phonosurgery later as a secondary procedure. In this study, all evaluated parameters in the phonosurgery group were improved significantly immediately after surgery. In the comparison between immediate RLN reconstruction and phonosurgeries, several parameters were significantly better in the phonosurgery group than in the reconstruction group at 1 month. This phenomenon could be attributed to the effects of VF adduction by AA and/or thyroplasty type I. On the other hand, at 6 and 12 months after surgery, some parameters in the reconstruction group were significantly better than those in the phonosurgery group. Improved muscle tension and VF position due to laryngeal muscle reinnervation led to these results. Regarding AA and thyroplasty type I, vocal function was improved by shifting the paralyzed VF medially (Figure 4). Therefore, we cannot prevent muscle atrophy and flaccidity with time by these surgeries, and the long-term vocal outcome is inconsistent. Although there are some reports on the effectiveness of RLN reconstruction procedures in patients who have previously undergone thyroid cancer surgery with RLN resection [40], the peripheral stump of the RLN may be buried in the cicatricial tissue and be difficult to reach for reconstruction. NMP transplantation is a method of inducing laryngeal muscle reinnervation with implantation of a nerve-muscle pedicle directly into the laryngeal muscle (Figure 3). We usually adopt NMP for RLNP caused by the previous thyroid cancer surgery. In our previous study on various causes of unilateral persistent RLNP, the effectiveness of NMP methods was improved gradually over long periods, and some parameters of NMP with AA were better than those of AA with or without thyroplasty type I at 24 months after surgery [26].

The limitations of this study were the small number of patients and the inconsistency of patient ages and sexes. Some parameters of vocal function are easily influenced by age and sex [41,42]. Therefore, further studies including sufficient number of patients who are age- and sex-matched are necessary.

## 5. Conclusions

For preoperative functioning RLN, the shaving technique contributes to maintaining vocal function and VF mobility. When the RLN cannot be resected to completely remove thyroid cancer, immediate RLN reconstruction with thyroid cancer surgery leads to excellent vocal outcomes. We can select any RLN reconstruction methods in such a situation, because their effectiveness is not associated with the surgical method, patient age, or preoperative RLNP. If it is difficult to perform immediate RLN reconstruction, it is desirable to perform phonosurgery as a secondary procedure, because postoperative vocal outcomes improve significantly compared to before surgery. Even in secondary surgery, it is preferable to select laryngeal reinnervation procedures, such as NMP with the AA method.

## Figures and Tables

**Table 1:** Patients’ profiles.

Factor	Reconstruction Group				Shaving Group	Phonosurgery Group
	Total	Direct Anastomosis	Nerve in Terposition	Nerve Transfer		
Number of patients	33	4	12	17	11	36
Sex (Male: Female)	9:24	0:4	5:7	4:13	2:9	23:13
Age range, year (median)	18–86 (64)	33–86 (54.5)	18–82 (66.5)	33–83 (64)	35–79 (63)	26–85 (59)
The period from onset to surgery, month (median)	1–12 (3)	0	1–12 (3)	1–14 (4)	-	0–445 (12.5)
Preoperative VF mobility (normal: paralysis)	12:21	4:0	4:8	4:13	11:0	0:36

VF: vocal fold.

**Table 2:** The average values of each parameters in paralyzed-reconstruction subgroup.

Subject	Preoperation	1 Month	6 Months	12 Months
MPT (s)	8.4 ± 5.6	8.5 ± 3.9	15.3 ± 8.0 **^†^	17.9 ± 8.7 **^††^
MFR (mL/s)	352 ± 280	356 ± 240	190 ± 111 ^††^	143 ± 55 *^††^
jitter (%)	4.217 ± 3.85	4.614 ± 3.69	1.576 ± 1.26 **^††^	1.161 ± 0.926 **^††^
shimmer (%)	10.463 ± 7.01	10.139 ± 5.47	5.422 ± 3.28 *^†^	5.560 ± 3.80 *^†^
HNR (dB)	6.867 ± 2.06	6.957 ± 2.36	8.376 ± 1.08 *	8.226 ± 0.952
G	1.54 ± 0.92	1.79 ± 0.68	0.67 ± 0.74 **^††^	0.41 ± 0.61 **^††^
B	1.29 ± 0.97	1.61 ± 0.73	0.53 ± 0.65 *^††^	0.20 ± 0.32 **^††^

MPT: maximum phonation time, MFR: mean airflow rate, HNR: harmonics-to-noise ratio; *: *p* < 0.05 compared with preoperation, **: *p* < 0.01 compared with preoperation. ^†^: *p* < 0.05 compared with 1month, ^††^: *p* < 0.01 compared with 1 month.

**Table 3:** *p* values of each parameters and periods in comparison between nerve interposition and nerve transfer subgroups in paralyzed-reconstruction subgroup.

Subject	Preoperation	1 Month	6 Months	12 Months
MPT	0.186	0.153	0.441	0.379
MFR	0.117	0.103	0.336	0.770
jitter	**0.013**	**0.014**	0.336	0.770
shimmer	**0.006**	**0.014**	0.054	0.057
HNR	**0.021**	**0.025**	0.773	0.143
G	0.099	0.813	0.643	0.426
B	0.163	0.453	0.474	0.676

MPT: maximum phonation time, MFR: mean airflow rate, HNR: harmonics-to-noise ratio; Bold characters mean nerve interposition subgroup were significantly better than nerve transfer subgroup.

**Table 4:** The average values of each parameters in phonosurgery group.

Subject	Preoperation	1 Month	6 Months	12 Months
MPT (s)	4.9 ± 2.6	14.2 ± 7.7 **	14.6 ± 7.2 **	13.9 ± 7.5 **
MFR (mL/s)	686 ± 423	242.2 ± 168.8 **	213.6 ± 145.3 **	227.7 ± 172.3 **
jitter (%)	9.010 ± 4.544	2.769 ± 3.069 **	3.195 ± 3.590 **	2.888 ± 3.020 **
shimmer (%)	15.199 ± 5.099	8.710 ± 6.605 **	10.063 ± 7.484 **	9.611 ± 7.701 **
HNR (dB)	4.375 ± 2.354	7.181 ± 2.041 **	6.796 ± 2.088 **	7.335 ± 1.942 **
G	2.28 ± 0.61	0.95 ± 0.76 **	0.83 ± 0.76 **	0.67 ± 0.56 **
B	2.15 ± 0.65	0.70 ± 0.73 **	0.67 ± 0.35 **	0.49 ± 0.44 **

MPT: maximum phonation time, MFR: mean airflow rate, HNR: harmonics-to-noise ratio. **: *p* < 0.01 compared with preoperation.

**Table 5:** *p* values of each parameter in comparison between shaving and normal-reconstruction subgroups.

Subject	12 Months
MPT	0.894
MFR	0.591
jitter	0.591
shimmer	0.884
HNR	0.961
G	0.576
B	0.760

MPT: maximum phonation time, MFR: mean airflow rate, HNR: harmonics-to-noise ratio.

**Table 6:** *p* values of each parameter and period in comparison between paralyzed-reconstruction subgroup and phonosurgery group.

Subject	Preoperation	1 Month	6 Months	12 Months
MPT	**0.009**	0.023	0.916	0.098
MFR	**<0.001**	0.049	0.490	0.112
jitter	**<0.001**	0.053	0.066	**0.006**
shimmer	**0.012**	0.259	**0.025**	**0.039**
HNR	**0.001**	0.920	**0.003**	0.117
G	**0.003**	0.003	0.412	0.164
B	**0.002**	0.003	0.280	0.057

MPT: maximum phonation time, MFR: mean airflow rate, HNR: harmonics-to-noise ratio. Bold character means reconstruction group was superior to phonosurgery group. Under line means phonosurgery group was superior to reconstruction group.

## Data Availability

The data presented in this study are available upon reasonable request.
